# A unique sonographic presentation of prenatal volvulus associated with malrotation

**DOI:** 10.1002/ccr3.4525

**Published:** 2021-07-06

**Authors:** Tamar Green, Daniela Chen, Tali Mishael, Ori Shen

**Affiliations:** ^1^ Department of Obstetrics and Gynecology Faculty of Medicine Shaare Zedek Medical Center Hebrew University of Jerusalem Jerusalem Israel; ^2^ Sackler School of Medicine Tel Aviv University Tel Aviv Israel

**Keywords:** malrotation, prenatal ultrasound, volvulus

## Abstract

This is a unique case of prenatal diagnosis of bowel malrotation suspected by an abnormal course of the duodenum. Early detection of volvulus was enabled, leading to timely intervention and a favorable outcome.

## Graphical abstract

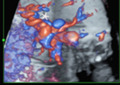

## 1 INTRODUCTION

A 36‐week gestation patient had an abnormal duodenum on sonography, suggesting malrotation. Volvulus was identified, prompting delivery and early intervention, resulting in bowel preservation. We found no previous reports in which a prenatal diagnosis of malrotation associated with volvulus was reported due to an abnormal duodenum and twisted mesenteric vessels.

Bowel malrotation, an uncommon spectrum of anomalies, is diagnosed after birth on upper gastrointestinal contrast radiography. Sonographic signs have limited sensitivity and include abnormal orientation of the superior mesenteric artery (SMA) and the superior mesenteric vein (SMV),^1^ and abnormal course of the third part of the duodenum.^2^ Prenatal diagnosis has been described in two cases^3^ due to midline position of the stomach. We found no cases in which the diagnosis was suspected on prenatal ultrasound due to an abnormal course of the duodenum or abnormal vessel layout.

Malrotation predisposes to midgut volvulus. The barber pole sign, representing twisting of the SMV around the SMA, is considered a specific sign of volvulus together with the whirlpool sign in which the dilated bowel loops spiral around the SMA. Nonspecific signs for volvulus include intestinal dilatation, differential fluid‐filled level in a dilated loop, polyhydramnios, meconium peritonitis, or pseudocyst.^4^

## 2 CASE REPORT

We report a unique case of prenatal diagnosis of evolving partial and atypical duodenal obstruction secondary to malrotation, and early identification of intrauterine midgut volvulus.

**FIGURE 1:**
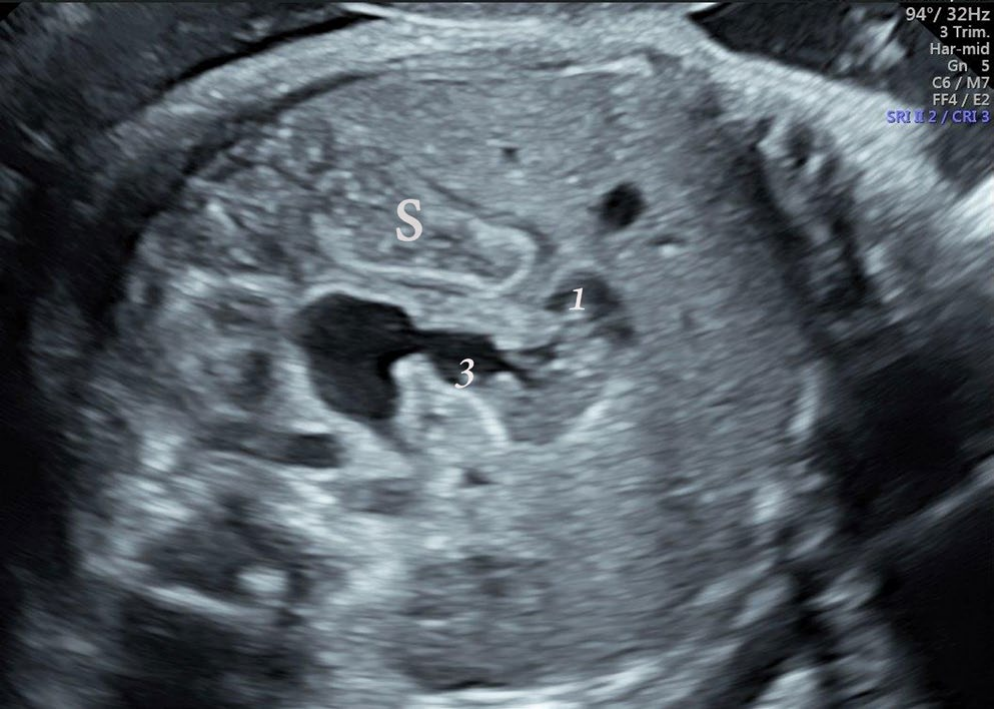
A 36‐week gestation. The abnormal course of mildly dilated duodenum suggesting malrotation. Duodenal segments marked 1 and 3, respectively. S, stomach

A 33‐year‐old woman, gravida 6 para 5, presented to our unit at 36+5 weeks gestational age because of a suspected abdominal cyst. On transabdominal sonography (GE Voluson E10, GE Medical Systems), an appropriately grown fetus with situs solitus, normal amount of amniotic fluid, and normal biophysical profile was seen. The stomach was full of echogenic fluid, with dilatation of all 4 parts of the duodenum, which took an abnormal course (Figure 1, video clip). It was not possible to confirm the SMA/SMV positioning, due to the fetal position. Malrotation was suspected due to the unusual course of the partially obstructed duodenum. No other abnormalities were evident. Fetal echocardiography was normal. The patient declined genetic amniocentesis and was asked to come in for follow‐up in 1 week.

**FIGURE 2:**
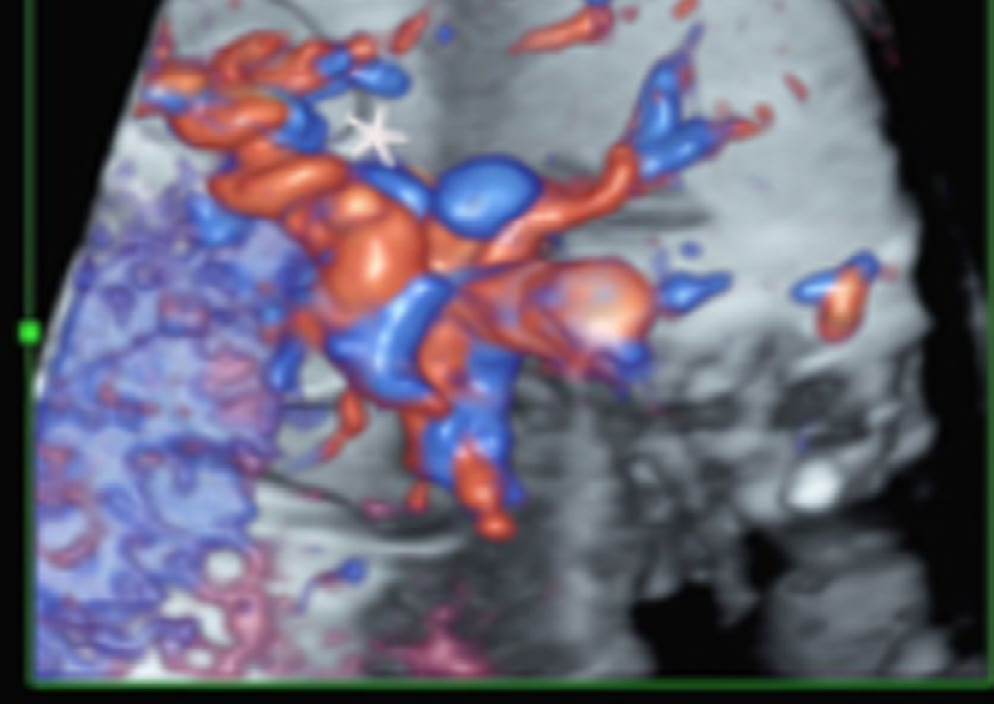
A 37‐week gestation. Midgut volvulus suggested by the "barber pole sign" with the twisted pedicle (*) of the superior mesenteric artery and vein

A week later, the duodenum was more severely dilated, with a normal amount of amniotic fluid. On power Doppler, an abnormal course of the SMA was seen corresponding to the clockwise barber pole sign (Figure 2), which raised the suspicion of midgut volvulus. Labor was induced medically, and the patient delivered within several hours a 2900 g girl with an Apgar score of 8/8. The neonate was asymptomatic. Due to the prenatal suspicion of malrotation and volvulus, the neonate had upper GI imaging and sonography, which were suggestive of malrotation and midgut volvulus. Intraoperative findings included 540‐degree volvulus of the midgut with malrotation. The Ladd procedure was performed with division of Ladd's bands, untwisting of the bowel, spreading of the mesentery, and straightening of the duodenum and appendectomy. The bowel was rearranged with the small bowel positioned on the right and the colon on the left. The bowel had normal color and consistency with only mild congestion of the mesentery without infarction or atresia.

## 3 DISCUSSION

The double bubble sign of proximal duodenal obstruction can be detected from the second trimester onward.^5^, ^6^ There is a high prevalence of associated structural anomalies (50%) and trisomy 21 (30%).^7^ The differential diagnosis includes other extrinsic or intrinsic causes for duodenal obstruction, for example, annular pancreas, intestinal malrotation with the Ladd bands, gastrointestinal duplication cysts, duodenal web, preduodenal portal vein, and choledochal cyst. A better prognosis may be expected when prenatal diagnosis is established. In this case, partial obstruction and the abnormal shape of the duodenum raised the suspicion of malrotation.

Intestinal rotation occurs in the first trimester. After having completed 270 degrees rotation from its original position, fixation of the normal position of the intestine occurs during the fourth and fifth months of gestation.^4^ In malrotation, this normal process fails to occur, locating the duodenal‐jejunal junction in the right upper quadrant, and the cecum in the middle‐to‐upper abdomen. Ladd's bands, typically present in malrotation, are fibrous peritoneal stalks that fix the cecum, in its abnormal location, to the gallbladder, duodenum, and right‐side of the abdominal wall. Consequently, malrotation predisposes the fetus or the child to midgut volvulus. Prenatal suspicion of malrotation should be considered in cases of heterotaxy, or when abnormal position of the stomach is noted on sonography.^3^ When asymptomatic malrotation is diagnosed, preventive Ladd's procedure is usually performed. In our literature search, we found no cases in which prenatal suspicion of malrotation was followed by a prenatal diagnosis of volvulus.

Prenatal manifestations of volvulus include decreased fetal movements, abnormal fetal heart rate, and fetal death.^8^ If left untreated, perforation of the lumen may occur with abdominal distention, hematochezia, pain, and irritability due to intestinal ischemia and peritonitis. Accurate prenatal diagnosis is crucial to prevent fetal death, to ensure a rapid and less complicated postnatal course, and to allow earlier evaluation of associated abnormalities and appropriate counseling.

In a series of eight cases^9^ of prenatal sonographic diagnosis of midgut volvulus, none had malrotation. Etiologies for volvulus include, other than malrotation, intestinal duplications,^10^ cystic fibrosis,^11^ and bowel atresia^12^ Specific sonographic signs included the whirlpool sign and the coffee bean sign. The former was seen in 2/8 cases and the latter in one. The other cases presented with nonspecific signs: dilated intestinal loops (8/8), polyhydramnios (3/8), ascites (2/8), gastric dilation (2/8), and pseudocysts (2/8). In a case of fetal midgut volvulus diagnosed with the whirlpool sign,^7^ the absence of a color Doppler signal corresponding to the barber pole sign was considered an ominous predictor for intestinal ischemia and impending perforation. In a recent series,^11^ 10/13 cases (77%) of prenatal volvulus, the whirlpool sign was visualized on sonography, while in the other three, only nonspecific signs were observed. None of these cases had malrotation.

Our case report illustrates a rare prenatal diagnosis of malrotation with the sonographic appearance of a partially obstructed duodenum and an abnormal course of its distal part. This was followed by intrauterine midgut volvulus, which led to immediate delivery and neonatal surgery, early enough to prevent damage to the intestine. It is difficult to determine the exact timing of the onset of volvulus, but this has likely occurred close to the onset of her follow‐up in our department. The detection of atypically positioned duodenum, with dilatation of its four parts, may be a manifestation of malrotation, a strong risk factor for volvulus. This should prompt a close follow‐up of fetal movements, fetal monitoring, and ultrasound to accurately determine the timing and mode of delivery.

## CONFLICT OF INTEREST

No conflict of interest is identified.

## AUTHOR CONTRIBUTION

Dr Green wrote the initial draft and reviewed subsequent ones. Dr Chen prepared literature report, collected and selected images, and reviewed final version. Dr Mishael, the clinician, managed the case. Dr Shen initiated the report, edited all versions, responded to editorial concerns, and is responsible for its accuracy and completeness.

## ETHICAL STATEMENT

I confirm that the patient consent has been signed and collected in accordance with the journal's patient consent policy. I will retain the consent form and will provide it if requested.

## Supporting information

Video S1

## Data Availability

Data available on request from the authors.
